# FDG-PET/CT and CT Findings of a Benign Solitary Fibrous Tumor of the Kidney; Correlation with Pathology

**Published:** 2015

**Authors:** Reiko Nakajima, Koichiro Abe, Tsunenori Kondo, Yoji Nagashima, Ken Kimura, Kenji Fukushima, Mitsuru Momose, Chisato Kondo, Kazunari Tanabe, Shuji Sakai

**Affiliations:** 1 Department of Diagnostic Imaging and Nuclear Medicine, Tokyo Women’s Medical University, Tokyo, Japan; 2 Department of Urology, Tokyo Women’s Medical University, Tokyo, Japan; 3 Department of Surgical Pathology, Tokyo Women’s Medical University, Tokyo, Japan

**Keywords:** Benign, FDG-PET/CT, Kidney, Solitary Fibrous Tumor

## Abstract

Herein, we report the F-18 fluorodeoxyglucose (^18^F-FDG) positron emission tomography (PET)/computed tomography (CT) findings of a benign solitary fibrous tumor (SFT) of the kidney. The patient was a 63-year-old woman with a mass in the right kidney (10×9.7 cm), incidentally found on CT images. The CT scan showed a lobulated tumor arising from the hilum of the right kidney. The tumor consisted of two components with different patterns of enhancement. Most of the tumor demonstrated moderate enhancement from the corticomedullary to nephrographic phase. A small nodular component at the caudal portion of the tumor showed avid enhancement in the corticomedullary phase and rapid washout in the nephrographic phase in contrast-enhanced CT. FDG-PET/CT was performed and showed weak FDG accumulation (SUV_max_=2.30 and 1.91 in the main and small caudal components). Although renal cell carcinoma was preoperatively diagnosed, histopathological examination revealed renal SFT, with no malignant potential. Therefore, when a renal tumor with contrast-medium enhancement and low FDG accumulation is demonstrated, SFT should be considered as a differential diagnosis in addition to renal cell carcinoma.

## Introduction

Solitary fibrous tumor (SFT) is a rare spindle cell neoplasm of mesenchymal origin, which may be either benign or malignant. SFT has been reported to arise from various organs, most frequently the pleura (1). Kidney is an uncommon site for SFT, and in the English literature, only 50 cases (approximately) have been reported (2–4).

Herein, we report the F-18 fluorodeoxyglucose (^18^F-FDG) positron emission tomography/computed tomography (PET/CT) findings of a benign renal SFT, which were correlated with the pathological results.

## Case report

**Figure 1:**
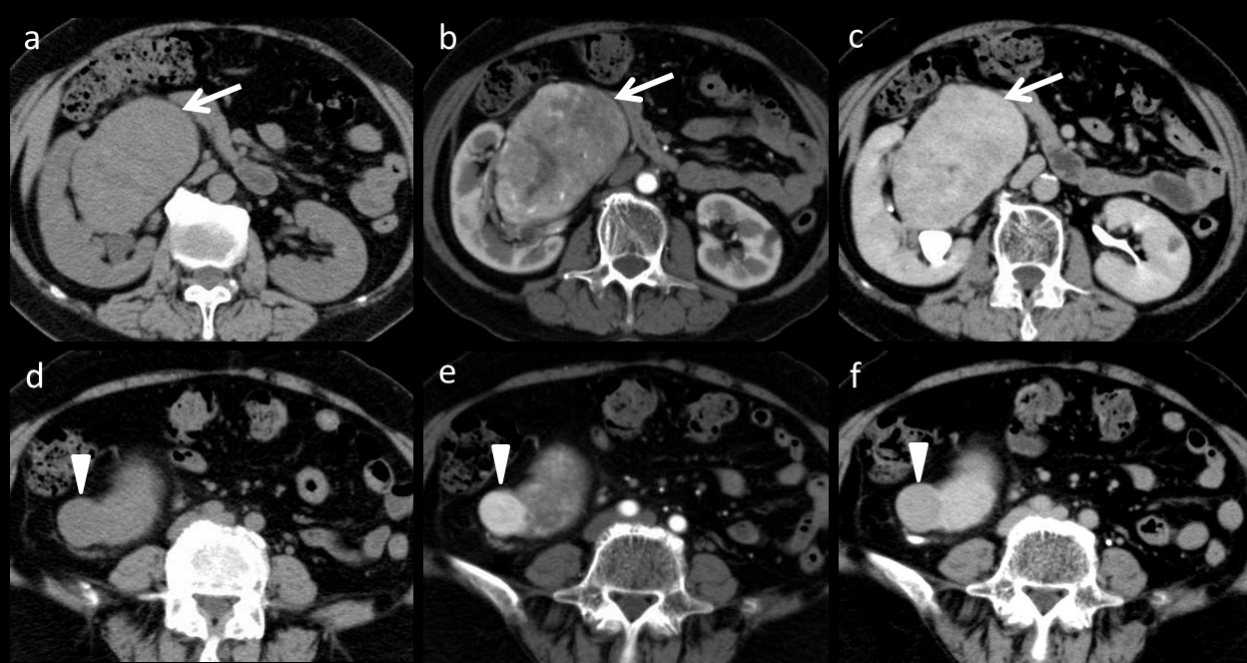
Axial non-contrast-enhanced CT image shows a large well-circumscribed lobulated tumor, arising from the hilum of the right kidney (a, d). The tumor consisted of two components with different patterns of enhancement. The corticomedullary (b) and nephrographic phases (c) showed moderate enhancement of the main part of the tumor. The small nodular component in the caudal portion of the tumor showed marked enhancement in the corticomedullary phase (e) and washout in the nephrographic phase (f)

A 63-year-old woman underwent CT scan for liver dysfunction. The CT images revealed a large, well-circumscribed, lobulated tumor, 10×9.7 cm in size (Figure 1), arising from the hilum of the right kidney. Most of the tumor demonstrated moderate contrast-medium enhancement from the corticomedullary to nephrographic phase (Figure 1b and 1c, arrows). There was a small nodular component bulging from the caudal portion of the tumor, showing avid enhancement in the corticomedullary phase (Figure 1e, arrowhead) and washed out in the nephrographic phase (Figure 1f, arrowhead).

**Figure 2:**
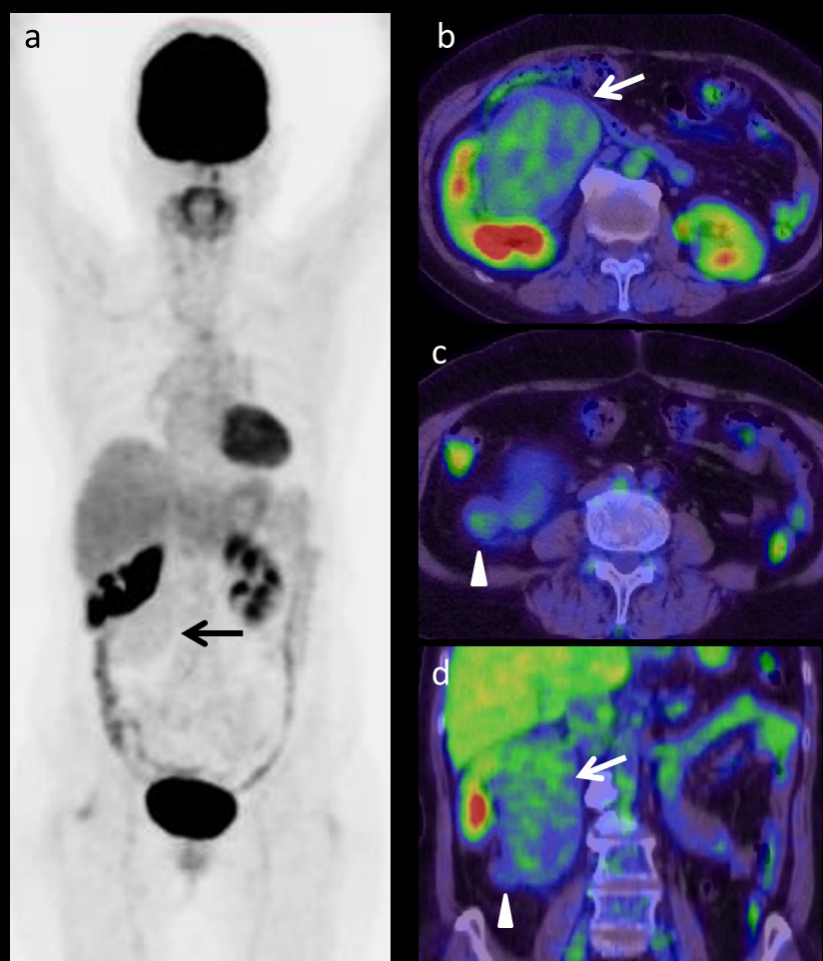
FDG-PET/CT showed low FDG accumulation in the renal tumor on maximal intensity projection (a) and axial (b, c) and coronal (d) PET/CT images. The SUV_max_ values of the main part and the nodular component of the caudal portion of the tumor were 2.3 (b, d, arrows) and 1.91 (c, d, arrowheads), respectively

Renal cell carcinoma (RCC) was initially suspected, and FDG-PET/CT was performed. The maximum intensity projection image showed weak FDG uptake, corresponding to the tumor (Figure 2a, arrow). FDG accumulation in the main and small caudal components was low with maximum standardized uptake values (SUV_max_) of 2.3 and 1.91, respectively (Figures 2b–d, arrows and arrowheads).

The degree of FDG uptake was similar in the two components of renal tumor, despite the difference in contrast enhancement. No abnormal FDG accumulation, suggesting a metastatic disease, was found elsewhere in the body.

**Figure 3:**
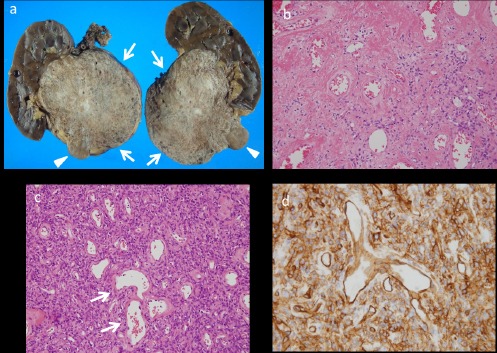
The cut surface of the tumor (10.5×9.5×7.5 cm) is presented in this figure. The main part of the tumor was yellowish white (arrows) and the small nodular component of the caudal portion of the tumor was grayish white (arrowhead). No evidence of either necrosis or hemorrhage was found (a). Light microscopic specimen showed tumor tissue with low cellularity, composed of spindle cells and thick-walled vessels haphazardly arranged with coarse collagen fibers in the main part of the tumor (b, H&E, original magnification ×10). The small nodular component of the caudal portion of the tumor was composed of tightly packed fusiform cells with numerous thin-walled ‘staghorn’ branching vessels (c, arrows, H&E, original magnification ×10). Immunohistochemically, the tumor cells were positive for CD34 (d, SAB method, original magnification ×20)

With a preoperative diagnosis of RCC, total nephrectomy was performed. The right kidney contained a well-circumscribed mass in the inferior portion (10.5×9.5×7.5 cm). The cut surface of most of the tumor was yellowish white (Figure 3a, arrows), and the small nodular component of the inferior region of the tumor was grayish white (Figure 3a, arrowheads).

Neither necrosis nor hemorrhage was observed in our case. Histologically, the former portion of the tumor was composed of various densities of fusiform or ovoid spindle-shaped cells and different amounts of large collagen bands with a patternless, storiform, or fascicular architecture (Figure 3b).

The small nodular component of the caudal part of the tumor, which showed intense enhancement and rapid washout in the contrast-enhanced CT, contained relatively dense spindle cells with a monotonous appearance and numerous thin-walled ‘staghorn’ branching vessels (Figure 3c).

Immunohistochemically, the tumor cells were positive for CD34 (Figure 3d) and negative for CD117, α-smooth muscle-specific actin, S-100 protein, cytokeratin (clone AE1/3), and melanosome-associated antigen (clone HMB45). The proliferating activity, assessed by Ki67 labeling index, was very low (less than 1%, data not presented). The tumor cells demonstrated no increased mitotic activity in the histological sections. Therefore, we diagnosed a benign renal SFT, without malignant potential. The patient is currently disease-free after 6 months of follow-up.

## Discussion

SFT is a rare neoplasm of mesenchymal origin, composed of bland spindle cells. It most commonly arises from the pleura, although it can develop from any organ in the body including the kidney (1). To the best of our knowledge, this is the first report of FDG-PET/CT findings of renal SFT.

Although SFT has been observed in patients of all ages, the majority of reported cases are older than 40 years. Renal SFT is a gross well-circumscribed slow-growing mass, arising from the renal parenchyma, renal capsule, cortex, or peripelvic connective tissue. This tumor may grow in size (with a diameter >100 mm), sometimes resulting in compression symptoms (5).

Microscopically, SFTs consist of fusiform or ovoid spindle cells with a patternless, fascicular, storiform or fibrosarcoma-like arrangement. Various amounts of hyalinized collagen bands have been observed in these tumors (6). Depending on the cellularity and the amount of collagenous fibers, two common variants of SFT have been described: the fibrous type (the most common) and the cellular type. The former type has an alternating presence of hypercellular or hypocellular⁄fibrous area. The latter shows a monotonous appearance with moderate to high cellular­ity, little intervening fibers, and numerous thin-walled ‘staghorn’ branching vessels.

SFT cells lack apparent features of differentiation and possess round-to-oval monomorphic nuclei, along with a pale, scanty cytoplasm. Also, the hemangiopericytoma- like pattern is a characteristic feature (6, 7).

Differential diagnoses were required from benign and malignant spindle cell neoplasms, i.e., leiomyomatous, schwannian neoplasms, so-called gastrointestinal stromal tumors (GISTs), and even from spindle cell liposarcoma and carcinoma. Immunohistochemically, tumor cells of SFTs failed to show obvious differentiation and were negative for myogenic (smooth muscle-specific actin and desmin), schwannain, and lipomatous neoplasms (S100 protein) and CD117 (c-KIT), a positive marker for GIST.

Characteristically, SFT cells demonstrate diffuse positive reaction to CD34 and bcl-2, which are now considered as SFT markers (6, 7), although not necessarily specific. Moreover, STAT6 has been recently known as a specific positive marker of SFT due to its NAB2-STAT6 gene fusion (8). As previously described, biological behaviors of SFTs are sometimes difficult to predict. Ki-67 and p53 labeling indices are generally low for benign SFTs (6).

In our patient, the larger component of the tumor showed weak FDG accumulation and moderate-to-high contrast enhancement in the corticomedullary phase. The small nodular component of the inferior part of the tumor showed washout in the nephrographic phase on contrast-enhanced CT images. Although these radiological findings suggested RCC, renal SFT was revealed by histological findings.

The microscopic examination revealed two distinct histologic appearances: the fibrous type and the cellular type in one tumor. The small nodular component of the tumor, which enhanced avidly in the corticomedullary phase and washed out in the nephrographic phase, was a cellular type. We believe that CT features reflected increased blood vessels, high cellularity, and less abundant collagenous fibers. The upper lesion, in contrast, was from the fibrous type, which contained dense collagen fibers with low cellularity. Therefore, in our patient, dynamic CT findings were attributable to the combination of variable cellularity, dense collagen content, and vascularity.

We also found that both components of renal tumor showed weak FDG accumulation on PET/CT scan, which did not positively indicate tumor malignancy. FDG-PET/CT has low sensitivity for the diagnosis of RCC, given the renal excretion of FDG (9). Therefore, the low SUV of renal tumor in our patient was not sufficient to exclude RCC. In fact, benign renal tumors cannot be easily distinguished from the malignant type, based on only FDG-PET/CT findings.

As several recent studies have revealed, FDG-PET/CT might be useful for the differentiation of benign and malignant SFTs of pleura and liver (10, 11). However, a few cases have demonstrated that the use of FDG-PET/CT for the differential diagnosis of benign and malignant SFTs may be potentially hazardous, and FDG-PET/CT cannot replace histology in this setting (12–14). Therefore, it is necessary to collect further information on the FDG-PET/CT findings of SFTs, including those of the kidney, to distinguish between malignant and benign tumors.

In conclusion, we reported a case of a benign renal SFT that was difficult to diagnose by preoperative clinical imaging. SFTs are characterized by a wide range of morphological features and can show various image findings. When a patient presents with a renal tumor with low FDG accumulation, SFT should be considered in addition to RCC. Familiarity with cross-sectional imaging findings may help clinicians make an accurate diagnosis.
